# Action Imitation Changes Perceptual Alternations in Binocular Rivalry

**DOI:** 10.1371/journal.pone.0098305

**Published:** 2014-05-28

**Authors:** Enrico Di Pace, Chiara Saracini

**Affiliations:** 1 Department of Psychology, “Sapienza” University, Rome, Italy; 2 IRCCS, Fondazione Santa Lucia, Rome, Italy; Barrow Neurological Institute, United States of America

## Abstract

Binocular rivalry is a visual phenomenon in which perception alternates between two different monocular images presented to each of the two eyes. Here, we propose using this phenomenon as a method to study the relation between action execution and action perception. In our experiment, a simple background (a checkerboard) was contrasted with a video representing a hand continuously grasping and releasing a ball. In Experiment 1, our subjects were asked to reproduce the perceived movement with their right hand whenever they became aware of it and to stop doing this when the checkerboard dominated. Our results revealed that motor imitation of the perceived action significantly increased the time spent perceiving the hand. Three control experiments showed that these effects were not due to a generic involvement of focused attention (Experiment 2 and 3), to a verbal description of the performed action (Experiment 3) or to the execution of an unrelated movement of the hand (Experiment 4). Although an intrinsic connection between action execution and attention cannot be excluded with certainty, and the boundary between action imitation and unrelated action execution may vary along various degrees of similarity, on the whole, the present results seem to suggest, at least on a preliminary basis, that action imitation do play a relevant role in the perception of action. We discuss these findings in the frame of current theories concerning the relation between perception and action.

## Introduction

When two conflicting images are presented to each eye using different techniques (e.g. a stereoscope, anaglyph spectacles or crossing the two eyes until the two images are superimposed), the two images spontaneously start to alternate every few seconds, rivaling each other for exclusive dominance in perceptual awareness.

This phenomenon, called binocular rivalry has been mainly studied in the attempt to locate its source in the multiple levels of the cognitive hierarchy [1]. Behavioural, fMRI and single-cell recording studies have suggested that low- [2]–[7] and high-level [8]–[17] adaptation throughout the visual system can have an equally influential, or even causal, role in controlling the effect.

More recently, binocular rivalry has been used to demonstrate that personality traits (e.g. general anxiety) [18]–[20], the positive or negative overt affective value of the rival images presented [21]–[23], and even learned affective information or “gossip” (personality traits contingently and randomly associated with completely unknown faces [24]) can influence the rate of the alternation of the perceived images and their relative period of dominance. In this perspective, the researcher's interest is not in discovering the structure of the phenomenon in itself, but rather in using it as a dependent variable, a sort of perceptual unit of measurement for quantifying how different variables may directly influence the way we select images and, more broadly, perceive the world.

The aim of the present study was to use binocular rivalry to investigate for the first time how imitating an action influences the perception of the same action representation.

The idea that perception and action are closely related and influence each other can be traced back to two different theoretical sources. From a perceptual perspective, the concept of ‘affordance’ [25] expresses the intuition that perception of the external world always entails information about potentially related actions.

From a more ‘action based’ perspective, the theory of ideomotor action [26] assumes that the anticipatory representation of an action's sensory feedback (which is in itself a sensory-response *image*) is used to control an action.

A closer and tighter combination and interplay between the perceptual and action dominions has been more recently formulated in a general theoretical framework that is known as the Theory of Event Code [27], [28], according to which, stimuli and responses are represented in the cognitive system in terms of an abstract common code. Evidence regarding this close (and) bi-directional link between perceptual and motor processes has been provided by a number of behavioural studies which show that an image can prime the motor system and speed up the initiation of an action (*visuomotor priming*) [29]–[32]. On the other hand, vision can be affected by action-induced effects [33]–[40]. In this case, the motor preparation of an action facilitates the recognition of a target image that is congruent with that action (*motor-visual priming*).

The recent discovery of neurons in both the monkey [41], [42] and human cerebral cortex whose activity is triggered either by the observation or execution of actions, seems to be the strongest evidence that the combination (integration) of perception and action actually occurs in the cerebral cortex. In general, it is believed that the IFG (Inferior Frontal Gyrus) [43], IPL (Inferior Parietal Lobe) [44] and STS (Superior Temporal Sulcus) form the neural circuit subserving what has been called the “Action Observation Network” (AON) [45]–[48] or “Motor Resonance System” [49].

Binocular rivalry could be a simple and powerful way to explore the relationship between action and conscious perception. Any variation in the pattern of dominance of the competing stimuli as a function of action execution should provide a direct measure of the influence that executing the action has on the perception of the stimulus related to that action. Accordingly, Maruya and colleagues [50] and Beets and colleagues [51] demonstrated that moving a cursor in the direction of a moving pattern makes that direction dominate for longer in the perception of rival moving patterns.

However, if one attempts to interpret the behavioural data in terms of the underlying neurophysiological structures, moving patterns do not seem to be the most suitable stimuli for achieving this goal, as one of the distinctive features of the AON is that it seems to be susceptible to biological motion, but not to motion in general [52], [53].

**Figure 1:**
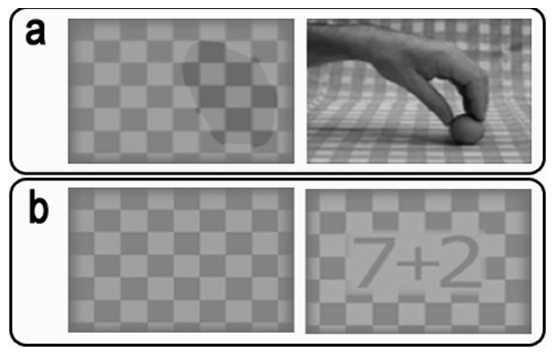
The rival stimuli used in the Experiments. (a) Stimuli used in experiments 1, 3 and 4: in the stereoscope mirror, the subjects actually saw a right hand continuously grasping and releasing a small ball. (b) The rival stimuli in experiment 2: in the original image, numbers were reversed and mirrored in the stereoscope so they were seen correctly by the subjects.

Here, we test the effect of action execution on the perception of the ***same*** action representation in a binocular rivalry condition. In the main experiment, we presented subjects with a pair of stimuli, one of which consisting of a neutral checkerboard and the other a video continuously repeating the sequence of a hand grasping and releasing a small ball (see Fig. 1a). We compared the duration of the alternations between the two stimuli in two conditions: in the first condition, the subjects passively observed the stimuli and simply reported which one dominated at any given time (“observation” condition); in the second, the subjects had to execute with their hand the movement they observed, but only when the image of the grasping hand was dominant (“task execution” condition).

Three further control experiments were planned in order to differentiate the possible effect of action imitation from other possible sources of influence, such as focused attention (experiments 2 and 3), verbal description of the action (experiment 3) and generic motor activation (experiment 4).

## Materials and Methods

### Participants

Forty participants with normal or corrected to normal vision took part in the study. There were 10 subjects (five women, 22±30 years) in the main experiment (Experiment 1), 10 (four women, 21–32 years) in the first control experiment (Experiment 2), 10 (six women, 20–30 years) in the second control experiment (Experiment 3) and 10 (four women, 22–32 years) in the third control experiment (Experiment 4). All the participants were students in the faculty of Psychology.

### Ethical statement

Written informed consent was obtained from all participants. The study protocol was approved by the local ethics committee (“Sapienza”, University of Rome) in accordance with the ethical standards of the Declaration of Helsinki.

### Materials

Our stereoscope was made in poliplat. A square hole (the ‘picture panel’) was located at each end of the stereoscope and allowed the subjects to view what was displayed at each end of the instrument. Each image was projected to a different eye by means of two mirrors located at the centre of the box, forming a 90° angle. Two holes were cut in the surface of the stereoscope in front of the vertex of the two mirrors to allow for the projection of the two images reflected by each mirror to both eyes. Two monitors, which were connected to two computers (running the Windows XP Operating System), were placed at the bottom ends of the stereoscope to project the two rival stimuli.

### Stimuli

In Experiments 1, 3 and 4, the rival stimuli were a checkerboard pattern image (we refer to this as the ‘motionless stimulus’) and a video showing a hand grasping and releasing a ball on a checkered background (we refer to this as the ‘dynamic stimulus’), as shown in Fig. 1a. Across experiments all stimuli had the same dimensions (i.e. each had a 3.45°×2.78° visual angle along the horizontal and vertical axes, respectively). As the hand introduced a clear element of discontinuity with respect to the background, we added a darker shadowy and motionless spot (which resembled the silhouette of the grabbing hand) to the checkerboard (neutral) stimulus. This shadowy silhouette was placed in the same position as the hand in the rival stimulus (see Fig 1a, left side). The averaged luminance of the checkerboard and the video stimuli were 37.3 and 23.8 cd/m^2^, respectively.

The rival stimuli in Experiment 2 consisted of: 1) the same checkerboard as in Experiment 1, but without the dark shadow (we also called this the ‘motionless stimulus’); 2) the two numbers ‘7’ and ‘2’, with an arithmetic operator in between (“dynamic stimulus”; see Fig. 1b). The two numbers were surrounded by a checkerboard frame. The arithmetic operator changed continuously every two seconds and consisted of one of four symbols: + (plus), − (minus), ÷ (divided), and × (multiplied). To prevent the subjects from becoming used to the answer sequence, the operator sequence changed in a quasi-random way. The average luminance value was 48.4 cd/m^2^ for the motionless stimulus, and 46.5 cd/m^2^ for the dynamic one.

### Procedure

The experimental session was organized in four blocks lasting five minutes each. Each block consisted of two conditions: 1) an “observation condition” in which the subjects had simply to observe the two stimuli in the stereoscope and report which one was dominant at any time; and 2) a “task execution condition” in which they had to perform a specific task on the “dynamic stimulus” for as long as they perceived it to be dominant (see the specific instructions for each experiment below). The order of the “observation” and “task execution” conditions was counterbalanced between the subjects.

The experimenter's instructions stressed the criteria the subjects had to use at all times to decide which stimulus was dominant. For example, for the “dynamic stimulus” to be considered dominant, its content had to be entirely visible (e.g. the hand performing the entire movement or the two numbers plus the arithmetic operator between them). Once the “dynamic stimulus” had gained access to conscious perception, the subjects were told not to switch to the other stimulus if only part of it was visible (e.g. a fragment of the checkerboard popping up somewhere in the image); instead, they had to wait until the checkerboard showed no further sign of the “dynamic stimulus”. Once the “motionless stimulus” had gained access to the subject's awareness, it had to be regarded as dominant even though clues regarding the other stimulus appeared somewhere in the frame (e.g. a finger or a type of moving transparent shadow).

All of the subjects' responses in the experimental sessions were recorded by a blinded collector-experimenter on a third computer using the Superlab 2.1 program. The collector-experimenter pressed one of two keys to record the subjects' responses. A second experimenter (one of the two authors, nearly always C.S.) was present in the lab during the experiment, in order to instruct the subjects and control that they conformed to the tasks requirements.

Before the experimental phase, each participant was trained to distinguish rival images and to express their perception at any particular time (think-aloud protocol). To this end, two grids were used which were composed of half of the image in vertical lines and the other half in horizontal lines. One grid was green and the other red. Accordingly, the subjects could perceive four patterns of dominance: one red image, one green image, one image consisting of vertical lines (half green and half red) and one consisting of horizontal lines (left side one colour, right side the other colour). Subjects who were unable to distinguish at least two of the different rival patterns did not take part in the real experiment. Subjects who correctly reported some sort of perceptual alternations performed a ‘pre-test’ version of the experimental procedure where they were presented for two minutes with the rival stimuli of the real experiment (see below for more details). During this period, the participants simply had to report which image was dominant at any given time, and their responses were recorded and immediately used as a further measure of sampling. Subjects who failed to report a balanced percentage of alternations between the two stimuli (not beyond the limit of 40% and 60%) did not take part in the rest of the experiment.

The following sections provide more specific information about the instructions, depending on which of the four experiments the subject was involved in.

#### Experiment 1

In the “task execution” condition, the subjects had to perform the action they observed (i.e. grasping and releasing a ball placed on a table in front of them, under the stereoscope and hidden from their view) whenever they perceived the hand to be the dominant stimulus. The participants had to verbally tell the experimenter which stimulus was dominant at any given time by saying ‘hand’ or ‘checkerboard’ for the “dynamic” and “motionless” stimuli, respectively. They were also told to pronounce the word only once at the point of transition, and to remain silent until the next transition. In the “task execution” condition, the subjects had to start to imitate the action performed by the perceived hand as soon as they indicated it was dominant, and only for as long as it was dominant. In other words, they had to stop reproducing the action as soon as the “motionless” stimulus started dominating.

#### Experiment 2

In this experiment the subjects had to say the word “checkerboard” or “numbers” as soon as either the “motionless” or the “dynamic” stimulus became dominant in their perception (here again, they had to pronounce the appropriate word only once at the moment of transition, and to remain silent until the next perceived change occurred). In the “task execution” condition, immediately after the “dynamic stimulus” became dominant, the subjects had to execute the operations indicated by the arithmetic operator between the two numbers and express the results out loud. The second experimenter checked that the subjects' responses were correct and provided feedback if they were not, while the blinded collector-experimenter independently continued to record the subjects' responses. This approach was utilized to ensure that the subject's attention was on the experimental task. No subject reported any difficulty while executing the task. Here again, the subjects had to stop as soon as the “motionless stimulus” was dominant.

#### Experiment 3

The “task execution” condition in the third experiment consisted of a verbal-action task. As in Experiment 1, the subjects had to say the word “hand” as soon as they saw that it was dominant in the stereoscope. Immediately after saying the word to the experimenter, the subjects had to start to verbally describe the action they were actually perceiving by saying “open”, “close”, “open”, “close” etc. as long as the perception of the hand continued; they were asked to stop speaking as soon as the motionless stimulus became dominant in their perception.

#### Experiment 4

In the “task execution” condition of Experiment 4, subjects were requested to execute with their hand an action which was unrelated to the one actually perceived in the stereoscope. They placed their hands on a table in front of them, under the stereoscope and hidden from their view. The palms of the subjects' hands were oriented upward. As in Experiment 1, the subjects had to say the word “hand” as soon as the corresponding stimulus became dominant in their perception. At the same time, they had to start moving in turn each finger of their right hand (which was always the same hand they saw in the stereoscope) upward and back to the starting position. We chose this movement because it was easy enough to be performed by the subjects and did not resemble in any respect the movement performed by the visual stimulus in the stereoscope. Moving each finger at a time prevented subjects from simulating (mentally or manually) any prehension movement. Furthermore, subjects were told not to simulate or reproduce any feature of the moving hand perceived in the stereoscope: i.e. they should not try to move their fingers at the same speed or with the same rhythm of the moving hand. Hand movement had to be stopped as soon as the “motionless stimulus” became dominant.

### Statistical analysis

To eliminate differences between the subjects and obtain a normalized value for the alternation durations, we transformed the raw data with the ratio (a−b)/(a+b), where “a” was the averaged dominance duration of the “dynamic stimulus” and “b” was the averaged dominance duration of the “motionless stimulus” for each condition/block and each subject. Positive values of this “dominance index” (DI) indicate that the “dynamic stimulus” (hand in Experiments 1, 3 and 4; numbers in Experiment 2, see below) prevailed in the competition against the “motionless” one. This “normalized” value of the dominance time (the DI) was the dependent variable in all of our analyses.

Mixed model 2×2×2 ANOVAs were performed separately for Experiments 1 and 2, with Condition (“observation”/”task execution”) and Block (first block/second block) as within factors and with Sequence (starting with the “observation condition” vs starting with the “task execution” one) as a between factor. To compare Experiments 1, 3 and 4 we performed a mixed model 3×2×2×2 ANOVA, with Experiment (1, 3 and 4) and Sequence as between factors and with Condition and Block as within factors.

As an index of size effect the eta-squared (ηp^2^) measure of variance was used [54]. The post-hoc pairwise comparisons were performed using the Bonferroni test. A significance threshold of P<0.05 was set for all of the statistical analyses. The data are reported as the mean ± standard error of the mean (SEM).

Data were analyzed using SPSS 21 software.

## Results

### Experiment 1

**Figure 2:**
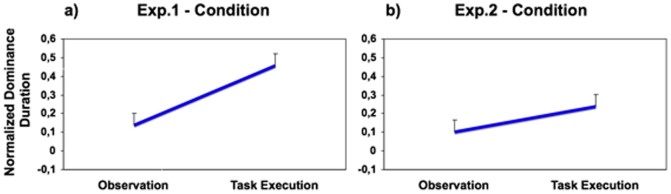
The effect of condition in Experiments 1 and 2. Panel (a) refers to Experiment 1, panel (b) refers to Experiment 2. The asterisk indicates significant effects (* = <0.01; ** = <0.005). Values reported on the Y axis express the difference between the mean alternation durations of the “dynamic” stimulus (the hand or the numbers) compared with the “motionless” one (the checkerboard); positive values express longer durations for the “dynamic” stimulus than the “motionless” one.

The analysis revealed a main effect of the Condition factor (F(1,8) = 18.483; p<0.001; ηp^2^ = 0.698): the “dynamic stimulus” (moving hand) dominated for longer durations in the “task execution condition” (DI mean: 0.455±0.069) compared to the “observation condition” (DI mean: 0.138±0.063; see Fig. 2a). No other main effect or interaction was significant.

As the results of the first experiment clearly revealed an increase in the dominance duration of the image of the performing hand in the “task execution condition” (action imitation), we ran three control experiments in order to explore more in depth three issues. The first one concerns the possible role of attention in the observed effect (Experiments 2 and 3), the second explores the possibility that the effect may be due to a representation of the action at an abstract level (Experiment 3) and the third compares the contribution of action imitation to the contribution of action execution in general (Experiment 4).

In Experiment 2, the “dynamic stimulus” consisted of the two numbers 7 and 2 with an arithmetic operator in between which changed every two seconds (see Fig. 1b). In order to catch subjects' attention, in the “task execution condition” they had to execute the operations indicated by the arithmetic operator as soon as it changed and express the results out loud.

### Experiment 2

The Condition effect was not significant here (p = 0.117, but ηp^2^ = 0.278, see Fig. 2b). Also in this case, the “dynamic stimulus” (DI mean: 0.238±0.066) tended to win the competition against the motionless stimulus in the “task execution condition” (DI mean: 0.101±0.050) but the effect was clearly not as strong as in Experiment 1. In contrast, the between subjects Sequence factor was significant (F(1,8) = 7.377; p<0.05; ηp^2^ = 0.480): the dynamic stimulus dominated for longer durations when the subjects started with the “task execution condition” (performing the computation; DI mean: 0.288±0.062) compared to when they started with the “observation condition” (simple observation; DI mean: 0.051±0.062). We will discuss the sequence effect in greater detail later. No other main effects or interactions were significant.

The results of Experiment 2 show that simply performing an operation over the “dynamic stimulus” does not generate in itself an attentional shift that can account for the effects reported in Experiment 1. However no direct comparison could be made between the size of the effects in the two experiments, as critical stimuli were different. Therefore, we performed Experiment 3 using the same pair of rival stimuli as in Experiment 1 (see Fig. 1a). In the “task execution condition”, instead of imitating the hand action, subjects were requested to verbally describe it by saying ‘open’, ‘close’, ‘open’, ‘close’ etc. as long as the perception of the hand remained dominant.

It is also possible that the dominance of the hand perception in the “task execution condition” of Experiment 1 was not specifically due to action imitation but more generally, to the unspecific activation of the motor system involved in the execution of the action. To verify this possibility we designed a fourth experiment in which the stimuli were the same as in experiment 1 and 3. In the “task execution condition”, subjects were required to move in turn each finger of their right hand as soon as the moving hand became dominant in their perception and to stop doing it as soon as the checkerboard gained access to their awareness (for further details see methods).

### Experiments 1-3-4

**Figure 3:**
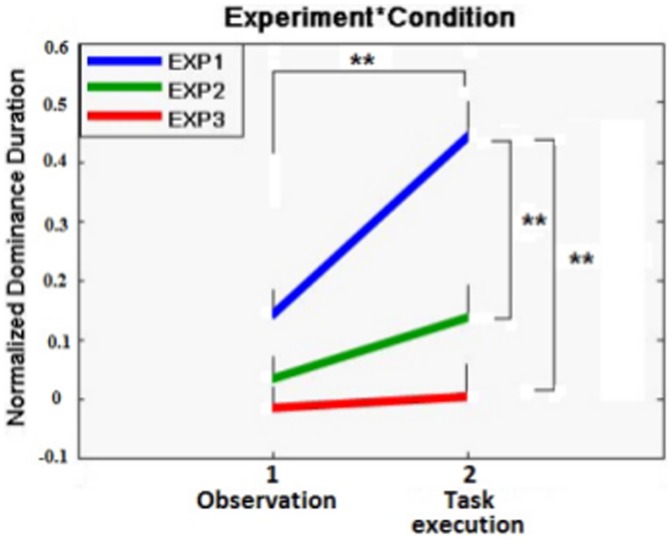
Experiment x Condition interaction in the combined analysis of Experiments 1, 3 and 4. The asterisk indicates significant effects (* = <0.01; ** = <0.005). The difference between the “task execution” condition and the “observation” condition is much larger in Experiment 1 compared to Experiments 3 and 4. Furthermore, the dominance durations of the “dynamic” stimulus (the moving hand) in the “task execution” condition were clearly much larger in experiment 1 (imitation) than experiment 3 (verbal description) and 4 (unrelated ation execution).

Experiment 3 and 4 were designed to be directly compared to Experiment 1. As a consequence we performed a mixed model 3×2×2×2 ANOVA with Experiment and Sequence as between factors and Condition and Block as within factors. The results showed a significant effect of the Condition factor (F(1, 24) = 18.886; p<0.001; ηp^2^ = 0.440), indicating longer duration times for the “dynamic stimulus” in the “task execution condition” (DI mean: 0.195±0.036) compared to the “observation condition” (DI mean: 0.049±0.026). The Experiment factor turned out to be significant as well (F(2, 24) = 11.739; p<0.001; ηp^2^ = 0.495). Dominance durations for the “dynamic stimulus” were longer in Experiment 1 compared to Experiments 3 and 4 (DI means: 0.297±0.046 vs 0.082±0.046 vs -0.012±0.046 for experiments 1, 3 and 4 respectively). Particularly relevant for the aim of this study was the presence of the Experiment x Condition interaction (F(2,24) = 7.191; p = 0.004; ηp^2^ = 0.375). In order to interpret the interaction, we performed multivariate tests to assess the influence of the Condition factor on each level of the Experiment factor, and univariate tests to assess the influence of the Experiment factor on each level of the Condition factor. As for the multivariate tests, the Condition factor showed an effect only in Experiment 1 (F(2, 24) = 29.888; p<0.001; ηp^2^ = 0.555). Bonferroni post-hoc pairwise comparisons showed that the “task execution condition” strongly differed from the “observation condition” (DI mean: 0.455±0.062 vs138±0.045, p<0.001; p<.001). The difference between the two conditions was much smaller in Experiment 3 (0.135±0.062 vs 0.030±0.045, p = 0.081) and null in Experiment 4 (−0.066±0.062 vs −0.019±0.045) (see Fig. 3 for a comparison between the two Conditions across the three Experiments).

The univariate tests assessing the influence of the three levels of the Experiment factor on each level of the Condition factor showed a significant effect only for the “task execution condition” (F(2,24) = 14.370; p<0.001; ηp^2^ = 0.545). Bonferroni post hoc pairwaise comparisons, showed that in the “task execution condition”, Experiment 1 strongly differed from experiment 3 (DI mean: 0.455±0.062 vs 0.135±0.062; p = .004) and Experiment 4 as well (DI mean: 0.455±0.062 vs −0.066±0.062; p<.001). (see Fig. 3 for a comparison between the three experiments in each of the two conditions).

Finally, the Sequence x Block x Condition interaction was also significant (F(1,24) = 8.552; p<0.01; ηp^2^ = 0.262). The interaction can be interpreted considering the univariate tests to assess the influence of the Condition factor on each level of the Block factor for each level of the Sequence factor. When the subjects started the experiment with an “observation condition” (Sequence 1) the difference beteween the “task execution” condition and the “observation” condition was significant but only in the first Block (F(1, 24) = 9.215; p = 0.006, ηp^2^ = 0.277; Block 1, “task execution” condition DI mean: 0.267±0.057, vs Block 1, “observation” condition DI mean: 0.106±0.031). On the other hand, when the subjects started the experiment with a “task execution” condition (Sequence 2), the reverse was true, in that the difference between the “task execution” and the “observation” conditions was relevant only in the second Block (F(1, 24) = 18.645; p<0.001, ηp^2^ = 0.437; the DI means for the “task execution” and “observation” conditions in the second Block were: 0.199±0.055 vs −0.041±0.050, respectively). No other test performed in order to interpret the interaction resulted significant.

**Figure 4:**
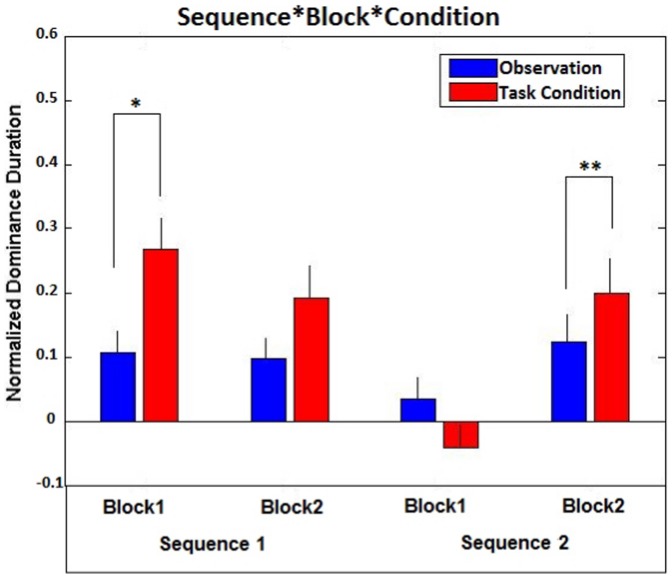
Sequence x Block x Condition interaction in the combined analysis of Experiments 1, 3 and 4. Blue bars refer to the “observation” condition, Red bars refer to the “task execution” condition (imitation in Experiment 1, verbal description in Experiment 3, unrelated action execution in Experiment 4). The asterisk indicates significant effects (* = <0.01; ** = <0.005).

As this interaction does not appear particularly relevant from the theoretical point of view, we will briefly discuss it now, putting off to the discussion section the widening of more relevant topics. When we were planning the experiment, we wondered whether a subject who started the experiment with a “task execution” condition (where he/she had to imitate the observed action or has to perform a calculation, in Experiment 2) would be induced to simulate the same action (only mentally) in the following “observation” condition. This is the reason why we decided to control for the Sequence factor. Indeed, it seems exactly what has occurred either in Experiment 2 and in the combined analysis of Experiments 1, 3 and 4. As shown in Fig. 4, when the subjects started with an “observation” condition the difference between the “task execution” and the “observation” conditions showed up; on the contrary, when the subjects started with a “task execution” condition, the same difference tended to disappear. However, this effect seems to vanish as a function of time and/or practice. In fact, in the second Block probably the subjects became accustomed to the experimental conditions, so that they become able to conform themselves more strictly to the experimental instructions, which required to simply observe in the “observation” condition and to imitate (or calculate, in Experiment 2) in the “task execution” condition. As a consequence, those subjects who started the experiment with a “task execution” condition, showed a difference between the “task execution” and the “observation” conditions only in the second Block. To sum up, it seems that starting with a “task execution” condition may result either in a general increase of the dominance durations of the “dynamic stimulus” (independent of conditions) as in Experiment 2 or in a general reduction of the difference between the two conditions (“observation” vs “task execution”) as in the combined analysis of Experiments 1, 3 and 4 (the two effects, being clearly related).

No other main effect or interaction proved significant.

## Discussion

In the present study, we used binocular rivalry to investigate the relationship between action imitation and action perception. For this purpose, we presented the subjects with a pair of stimuli, one representing a neutral checkerboard and the other consisting of a video that continuously reproduced a hand performing the action of grasping and releasing a small ball. Then, we compared the duration of the alternations between the two stimuli in two conditions: one in which the subjects passively observed the stimuli and simply reported which was dominant at any time, and the other in which the subjects had to perform with their hand the same movement that they were seeing, but only when the stimulus representing the grasping hand was dominant.

Results showed that when the subjects had to imitate the action, the stimulus representing that action remained dominant for longer durations compared to the condition in which they simply had to observe it. This finding is consistent with the idea that the system involved in action execution is also involved in promoting the conscious perception of the same actions.

Three control experiments were carried out in order to check the possible contribution that either a generic attentional bias, or a representation of the action at an abstract level, or a generic activation of the motor system could have exerted in the observed effect. Both attentional bias and abstract representation seemed to affect the perceptual alternations between the rivalling stimuli but neither seemed able to account for the size of the effects found in Experiment 1. In our opinion, the present results indicate that action imitation might play a relevant role, if not unique at least privileged, in the elaboration of stimuli which represent actions.

We will now discuss these results in more detail with reference to some theoretical issues recently discussed in the literature concerning binocular rivalry and the relationship between perception and action.

### Action imitation and binocular rivalry

To the best of our knowledge this is the first study on the relationship between action imitation and action perception in binocular rivalry. However we are by no means the first to explore the relationship between action and perception using binocular rivalry or bistable stimuli. The idea that eye movements have a role in the pattern of alternations in binocular rivalry or bistable perceptions dates back to the nineteenth century [55], [56]. Reflexive eye movements, like optokinetic nystagmus (OKN), have been used to monitor dominance in binocular rivalry [57], [58], [59] and have been found to be modulated by the perception of ambiguous motion [60]. Recently, a few studies explored the relationship between action and alternation patterns in binocular rivalry [50] and in bistable or ambiguous perceptions [51], [61]–[63]. In general, the results support the conclusion that the direction of a movement shapes the perceived direction of a bistable or ambiguous motion stimulus. By contrast, our stimuli and experimental design have the advantage of being related to a much more specific interpretation in terms of the neural substrates supposedly involved in the cross-talk between action and perception. Indeed, we interpreted our data in relation to specific involvement of the so-called “action-observation network”. Monkey studies indicate that a portion of the neurons in these regions implement a mirror mechanism, whose specific function is to match perceived actions with motor representations of similar actions [64] (for a study showing the crucial role of the mirror system on imitation in humans see [65]). By contrast, the relationship between moving patterns and homologous movements of the hand in the same or different directions seems to be a vaguer concept and more difficult to locate in terms of brain structures. We tried to assess more specifically the role of action imitation in the observed effects, by comparing a condition in which the subjects had to imitate the observed action with a condition in which the subjects activated the same effector system performing an action which was however unrelated to the perceived one (Experiment 1 vs Experiment 4) (We designed Experiment 4 in response to an objection raised by an anonymous reviewer. His/her suggestions helped us to provide stronger arguments in the attempt to specify the role of action imitation in the present results). Results clearly showed that only action imitation affected the pattern of perceptual alternations, while the simple activation of the motor system turned out to be ineffective. Of course the role of action imitation in shaping the perceptual alternations in binocular rivalry can be specified at a finer grain level. For example, action imitation could be performed by the same hand or by the opposite hand; the observed hand could be presented in different orientation which, in turn, could be related or orthogonal to the orientation of the performing hand; the action of the observed hand could be reproduced synchronously or asynchronously and so on. The anatomical correspondence between the observed and the performing effector [46], [66], as well as the congruency of their orientation [32], [35], [67], and the role of synchronicity between the reproduced and the observed action [40], are all factors which have been shown to influence the way in which the action is perceived or processed by the visual system. Thus, although we have not yet defined the full range of characteristics which can modulate the role of action imitation in binocular rivalry, we have shown that not any motor activation is able to exert the same effect. Simply activating the motor system during the perception of an action is not sufficient in order to affect the way in which that action is perceived.

### Motor code vs abstract code in action execution

When a subject perceives an action and reproduces it, the intention to move may be formulated in an abstract-conceptual code representing the content of the movement to be executed. Indeed, it has recently been proposed that the motor system may also be involved in the comprehension of abstract sentences related to actions [68]–[70]. However, data from experiment 3 clearly show that a verbal description of a perceived action is not as effective in shaping the pattern of alternation in binocular rivalry as imitating/performing that action. This evidence is congruent with one of the earliest theoretical formulations of the idea of a link between perception and action (Greenwald's theory of ideomotor action [26]). According to Greenwald, the execution of an action is controlled by an anticipatory representation of the action's sensory feedback. The more the format of the action representation is similar to the one used for action execution, the more the anticipatory representation will be transformed into the corresponding action (ideomotor compatibility) [71], [72]. Of course, when the observed action matches the required action, the level of similarity is maximal; in cases in which only verbal or symbolic descriptions are involved the level of similarity is lower.

### Motor code vs sensory feedback

The idea that some sort of sensory feedback may have a role in the effects of action execution over the pattern of alternations in the present experiment is indirectly supported by recent findings provided by studies which explored cross-modal interactions in binocular rivalry. These studies showed that touch [73], [74], sound [75], and even olfaction [76] may affect the pattern of alternation and dominance in visual binocular rivalry. From a cross-modal perspective, visuo-tactile integration may have affected our results in that during the action imitation the subject's fingers touched the ball. However, we think that the contribution of tactile sensory feedback can hardly be considered relevant in the present case. Suppose that we used a ball and a checkerboard as rival stimuli. In the attempt to isolate the contribution of tactile sensory feedback there would be a condition in which the subject's fingers are stimulated by a ball surface with no action to be imitated or performed. In that case it would be possible that the visual dominance durations of the ball would gain from the simultaneous availability of the congruent tactile sensory information. This does not seem to apply to our condition: there, the subjects instructions stressed the movement of the hand and the same held true as for the subjects' effort when they had to reproduce the action. It seems unlikely that the entire nature of the observed effect might be attributable only to the unattended and secondary effect of the sensation of the fingers touching the ball during the intentional execution of the action. (We are grateful to an anonymous reviewer for giving us the opportunity to explain our opinion on this topic).

In a more indirect way, a link between action execution and cross-modal integration in binocular rivalry has been recently suggested [51]. From this perspective, as one of the signals accompanying the action during motor execution consists of somatosensory re-afference signals, the influence of action execution over binocular rivalry may be interpreted in terms of a more general model of common integration among different sensory modalities [51]. As reafferentation is not required in the verbal description of a perceived action, it may be responsible for the specific improvements in action execution over binocular rivalry. Empirical evidence supporting the contribution of proprioceptive feed-back on binocular rivalry, has been recently provided by Salomon and colleagues, using a Continuous Flash Suppression paradigm [67]

Although the reafferentation hypothesis seems to be a good candidate in the attempt to explain the pattern of alternations observed in the present experiment, it cannot be taken for granted. It has been claimed that action can influence perception only when there is a functional relationship between them, i.e., when the integration of both is needed to perform a task, otherwise the effects are absent. In this respect, action planning in relation to the stimulus seems crucial to induce binding between action and perception [77]. As a consequence, it seems unlikely, at least at first sight, that all that matters in the intentional execution of the action performed in the present experiment, is the contribution of the reafferent signal. Such a contribution would have been present even though the action had been performed in a purely passive way.

However, we believe that this issue is an interesting one, deserving further and specifically dedicated empirical investigation.

### Motor code vs attention

Attention may influence Binocular rivalry in various ways, i.e by triggering rivalry [78], shaping the rate of alternations [79], [80], determining which of the two stimuli will become dominant first [81]–[83], reducing susceptibility to spontaneous switches [14], [81], prolonging perceptual dominance [79], [84] and enhancing the strength of the suppressed stimuli [85], [86]. We investigated whether attention has a role in modulating the pattern of alternations in binocular rivalry in our control experiments. In Experiment 2, we changed the stimulus competing with the checkerboard, showing to the subject an image representing the numbers 7 and 2 separated by an arithmetic operator randomly changing in time. In the “task execution” condition, the subject's task was to perform the operation whenever the stimulus containing the numbers was dominant. In Experiment 3, the stimuli were the same as in Experiment 1 but the task was to verbally reproduce the action represented by the hand in the rival video, again when the “dynamic” stimulus (hand) was dominant. Although these manipulations affected the pattern of alternations in Experiments 2 and 3, the effects were much smaller than in Experiment 1, when subjects had to actually imitate the perceived action. Can this result be interpreted by simply assuming that action imitation implies a stronger attentional bias/involvement towards the perceived stimulus? In the case of Experiment 2, the answer can be affirmative. In fact, Chong and colleagues [84] showed that in challenging visual tasks, focusing attention on the visual features of one of the two rival stimuli is required in order to change the alternation pattern. It may well be that in Experiment 2 subjects focussed on the arithmetic operator in order to perform the calculations and disregarded the two numbers, which were kept constant in the stimulus. This may have reduced the potential increase of the dominance durations for that stimulus, considered as a whole (the numbers ***and*** the arithmetic operator). However, the same criticism does not seem to hold for Experiment 3, since the stimuli were exactly the same as those in experiment 1 and to perform the task the subjects had to pay the same kind of attention to the visual features of the stimulus. There seems to be no obvious reason why moving a hand in synchrony with a perceived hand should be more attentionally demanding than saying words that describe the movement in synchrony with it. Therefore, we are more inclined to believe that our data confirm the idea that attention can modulate the phenomenon of binocular rivalry, but the modulation we report for action imitation leads to a further component probably related to the specific role of the motor system in perceiving stimuli representing actions. However, our conclusion cannot be certainly considered definitive. (We are grateful to an anonymous reviewer: his/her comments on this issue helped us to discuss more appropriately the possible role of attention in the present work). Imagine as an example a theoretical framework known as the “premotor theory of attention” [87]. This theory assumes that planning a goal directed action with any effector system is sufficient to trigger a shift in spatial attention. As a consequence, there would be no question about the possibility of keeping attention and motor planning apart. In this perspective, our conclusion should be restated as follows: being intrinsically connected to the attentional system, action imitation plays a special role in modulating the attentional bias toward the stimulus which represents the action to be imitated. It is this joint contribution of the motor and attentional systems (which could be even considered as a unified motor-attentional system) which affects the pattern of alternations in binocular rivalry.

The premotor theory of attention is an influential and yet disputed theory (for a recent review on this topic, see [88]). Thus it seems that the question of whether action imitation is intrinsically associated to attentional involvement is an interesting question that still remains to be answered. The present research project was not designed to specifically investigate this issue.

### Motor code vs volitional control

Although the question about the role of voluntary control over binocular rivalry seems to be homologous to that of the role of attention, the two topics are conceptually separate. Attention may contribute to selecting the dominant image, but once it has been selected, voluntary control should be needed to keep the image dominant. Particularly relevant for the topic of this paper is a work recently published on this journal [57]. The authors found that human observers can exert a significant amount of volitional control over competing stimuli in binocular rivalry, but only when the elaboration of the represented stimuli involves activation of the dorsal visual stream. Stimuli consisted of moving patterns or analogous configurations that represented apparent motions. Subjects were able to voluntary select the desired stimuli with respect to the competing one, but they were unable to do the same when the stimuli represented static patterns [57]. This finding is consistent with a previous neuroimaging report which showed that in a binocular rivalry stimulation condition, the dorsal stream was activated even when the stimulus representing a manipulable object tool was suppressed by the rival stimulus. On the contrary, stimuli like human faces activated the ventral stream only when they were dominant in the observers' perception [89]. Therefore, the idea is that the representation of moving patterns or stimuli linked to actions is always potentially available in the brain, so the subjects can select it whenever they want.

At first sight, this explanation seems to apply also to the present data, since the larger effect of experimental manipulations over binocular rivalry was obtained in a condition in which the stimuli directly represented execution of an action (a moving hand). But this interpretation can only partially account for the results, because even though the stimuli were exactly the same in experiments 1 and 3, the duration of the alternations varied to a greater extent in experiment 1 than experiment 3. Here again, the variation of the pattern of alternations seems to be due to a specific factor, which, in our opinion, is the direct involvement of the motor system in the perception of stimuli representing actions.

## References

[1] Tong F, Meng M, Blake R (2006) Neural bases of binocular rivalry. Trends Cogn Sci 10: 502–511. 16997612 10.1016/j.tics.2006.09.003

[2] Blake R, Westendorf DH, Overton R (1980) What is suppressed during binocular rivalry? Perception 9: 223–231. 7375329 10.1068/p090223

[3] Lee SH, Blake R (2004) A fresh look at interocular grouping during binocular rivalry. Vis Res 44: 983–991. 15031091 10.1016/j.visres.2003.12.007

[4] Meng M, Tong F (2004) Can attention selectively bias bistable perception? Differences between binocular rivalry and ambiguous figures. J Vis 4: 539–551. 15330700 10.1167/4.7.2 PMC1403736

[5] Polonsky A, Blake R, Braun J, Heeger DJ (2000) Neuronal activity in human primary visual cortex correlates with perception during binocular rivalry. Nat Neurosci 3: 1153–1159. 11036274 10.1038/80676

[6] Tong F, Engel SA (2001) Interocular rivalry revealed in the human blind-spot representation. Nature 411: 195–199. 11346796 10.1038/35075583

[7] Haynes JD, Rees G (2005) Predicting the stream of consciousness from activity in human visual cortex. Curr Biol 15: 1301–1307. 16051174 10.1016/j.cub.2005.06.026

[8] Carney T, Shadlen M, Switkes E (1987) Parallel processing of motion and colour information. Nature 328: 647–649. 3614368 10.1038/328647a0

[9] Kovacs I, Papathomas TV, Yang M, Feher A (1996) When the brain changes its mind: interocular grouping during binocular rivalry. Proc Natl Acad Sci U S A 93: 15508–15511. 8986842 10.1073/pnas.93.26.15508 PMC26435

[10] Logothetis NK, Leopold DA, Sheinberg DL (1996) What is rivalling during binocular rivalry? Nature 380, 621–624. 10.1038/380621a0 8602261

[11] Carlson T, He S (2000) Visible binocular beats from invisible monocular stimuli during binocular rivalry. Curr Biol 10: 1055–1058. 10996073 10.1016/s0960-9822(00)00672-2

[12] Pearson J, Clifford CWG (2005) Suppressed Patterns Alter Vision during Binocular Rivalry. Curr Biol 15: 2142–2148. 16332541 10.1016/j.cub.2005.10.066

[13] Blake R, Tadin D, Sobel KV, Raissian TA, Chong SC (2006) Strength of early visual adaptation depends on visual awareness. Proc Natl Acad Sci U S A 103: 4783–4788. 16537384 10.1073/pnas.0509634103 PMC1400587

[14] Lumer ED, Friston KJ, Rees G (1998) Neural correlates of perceptual rivalry in the human brain. Science 280: 1930–1934. 9632390 10.1126/science.280.5371.1930

[15] Tong F, Nakayama K, Vaughan JT, Kanwisher N (1998) Binocular rivalry and visual awareness in human extrastriate cortex. Neuron 21: 753–759. 9808462 10.1016/s0896-6273(00)80592-9

[16] Williams MA, Morris AP, McGlone F, Abbott DF, Mattingley JB (2004) Amygdala responses to fearful and happy facial expressions under conditions of binocular suppression. J Neurosci 24: 2898–2904. 15044528 10.1523/JNEUROSCI.4977-03.2004 PMC6729857

[17] Leopold DA, Logothetis NK (1996) Activity changes in early visual cortex reflect monkeys' percepts during binocular rivalry. Nature 379: 549–553. 8596635 10.1038/379549a0

[18] Alpers GW, Gerdes AB (2007) Here is looking at you: emotional faces predominate in binocular rivalry. Emotion 7: 495–506. 17683206 10.1037/1528-3542.7.3.495

[19] Nagamine M, Yoshino A, Yamazaki M, Obara M, Sato S, et al (2007) Accelerated binocular rivalry with anxious personality. Physiol Behav 91: 161–165. 17433385 10.1016/j.physbeh.2007.02.016

[20] Yoon KL, Hong SW, Joormann J, Kang P (2009) Perception of facial expressions of emotion during binocular rivalry. Emotion 9: 172–182. 19348530 10.1037/a0014714

[21] Alpers GW, Ruhleder M, Walz N, Muhlberger A, Pauli P (2005) Binocular rivalry between emotional and neutral stimuli: A validation using fear conditioning and EEG. Int J Psychophysiol 57: 25–32. 15893834 10.1016/j.ijpsycho.2005.01.008

[22] Bannerman RL, Milders M, De Gelder B, Sahraie A (2008) Influence of emotional facial expressions on binocular rivalry. Ophthal Physiol Opt 28: 317–326. 10.1111/j.1475-1313.2008.00568.x 18565087

[23] Anderson E, Siegel EH, Barrett LF (2011) What you feel influences what you see: The role of affective feelings in resolving binocular rivalry. J Exp Soc Psychol 47: 856–860. 21789027 10.1016/j.jesp.2011.02.009 PMC3141576

[24] Anderson E, Siegel EH, Bliss-Moreau E, Barrett LF (2011) The visual impact of gossip. Science 332: 1446–1448. 21596956 10.1126/science.1201574 PMC3141574

[25] Gibson JJ (1986) The ecological approach to visual perception. Hillsdale, NJ: Erlbaum.

[26] Greenwald AG (1970a) Sensory feedback mechanisms in performance control: With special reference to the ideomotor mechanism. Psychol Rev 77: 73–99. 5454129 10.1037/h0028689

[27] Prinz W (1997) Perception and action planning. Eur J Cogn Psychol 9: 129–154.

[28] Hommel B, Müsseler J, Aschersleben G, Prinz W (2001) The theory of event coding (TEC): A framework for perception and action planning. Behav Brain Sci 24: 849–878. 12239891 10.1017/s0140525x01000103

[29] Craighero L, Fadiga L, Umiltà CA, Rizzolatti G (1996) Evidence for visuomotor priming effect. Neuroreport 8: 347–349. 9051808 10.1097/00001756-199612200-00068

[30] Craighero L, Fadiga L, Rizzolatti G, Umiltà CA (1998) Visuomotor Priming. Vis Cog 5: 109–125. 10.1097/00001756-199612200-00068 9051808

[31] Tucker M, Ellis R (1998) On the relations between seen objects and components of potential actions. J Exp Psychol HPP 24: 830–846. 10.1037//0096-1523.24.3.830 9627419

[32] Vogt S, Taylor P, Hopkins B (2003) Visuomotor priming by pictures of hand postures: perspective matters. Neuropsychologia 41: 941–951. 12667530 10.1016/s0028-3932(02)00319-6

[33] Müsseler J, Hommel B (1997) Blindness to response-compatible stimuli. J Exp Psychol HPP 23: 861–872. 10.1037//0096-1523.23.3.861 9180047

[34] Craighero L, Fadiga L, Rizzolatti G, Umiltà CA (1999) Action for perception: a motor-visual attentional effect. J Exp Psychol HPP 25: 1673–1692. 10.1037//0096-1523.25.6.1673 10641315

[35] Craighero L, Bello A, Fadiga L, Rizzolatti G (2002) Hand action preparation influences the responses to hand pictures. Neuropsychologia 40: 492–502. 11749979 10.1016/s0028-3932(01)00134-8

[36] Bekkering H, Pratt J (2004) Object-based processes in the planning of goal-directed hand movements. Q J Exp Psychol A 57: 1345–1368. 15513250 10.1080/02724980343000765

[37] Casile A, Giese MA (2006) Nonvisual motor training influences biological motion perception. Curr Biol 16: 69–74. 16401424 10.1016/j.cub.2005.10.071

[38] Fagioli S, Hommel B, Schubotz RI (2007) Intentional control of attention: action planning primes action-related stimulus dimensions. Psychol Res 71: 22–29. 16317565 10.1007/s00426-005-0033-3

[39] Lindemann O, Bekkering H (2009) Object manipulation and motion perception: evidence of an influence of action planning on visual processing. J Exp Psychol HPP 35: 1062–1071. 10.1037/a0015023 19653749

[40] Christensen A, Ilg W, Giese MA (2011) Spatiotemporal tuning of the facilitation of biological motion perception by concurrent motor execution. J Neurosci 31: 3493–3499. 21368061 10.1523/JNEUROSCI.4277-10.2011 PMC6623932

[41] di Pellegrino G, Fadiga L, Fogassi L, Gallese V, Rizzolatti G (1992) Understanding motor events: a neurophysiological study. Exp Brain Res 91: 176–180. 1301372 10.1007/BF00230027

[42] Gallese V, Fadiga L, Fogassi L, Rizzolatti G (1996) Action recognition in the premotor cortex. Brain 119: 593–609. 8800951 10.1093/brain/119.2.593

[43] Chong TTJ, Cunnington R, Williams MA, Kanwisher N, Mattingley JB (2008) fMRI adaptation reveals mirror neurons in human inferior parietal cortex. Curr Biol 18: 1576–1580. 18948009 10.1016/j.cub.2008.08.068 PMC2766090

[44] Kilner MJ, Neal A, Weiskopf N, Friston KJ, Frith CD (2009) Evidence of Mirror Neurons in Human Inferior Frontal Gyrus. J Neurosci 29: 10153–10159. 19675249 10.1523/JNEUROSCI.2668-09.2009 PMC2788150

[45] Grafton ST (2009) Embodied cognition and the simulation of action to understand others. Ann N Y Acad Sci 1156: 97–117. 19338505 10.1111/j.1749-6632.2009.04425.x

[46] Iacoboni M, Koski LM, Brass M, Bekkering H, Woods RP, et al (2001) Reafferent copies of imitated actions in the right superior temporal cortex. Proc Natl Acad Sci U S A 98: 13995–13999. 11717457 10.1073/pnas.241474598 PMC61155

[47] Buccino G, Binkofski F, Riggio L (2004) The mirror neuron system and action recognition. Brain Lang 89: 370–376. 15068920 10.1016/S0093-934X(03)00356-0

[48] Avenanti A, Annella L, Candidi M, Urgesi C, Aglioti SM (2013) Compensatory Plasticity in the Action Observation Network: Virtual Lesions of STS Enhance Anticipatory Simulation of Seen Actions. Cereb Cortex 23: 570–580. 22426335 10.1093/cercor/bhs040

[49] Aglioti SM, Cesari P, Romani M, Urgesi C (2008) Action anticipation and motor resonance in elite basketball players. Nat Neurosci 11: 1109–1116. 19160510 10.1038/nn.2182

[50] Maruya K, Yang E, Blake R (2007) Voluntary action influences visual competition. Psychol Sci 18: 1090–1098. 18031417 10.1111/j.1467-9280.2007.02030.x

[51] Beets IAM, ’t Hart BM, Rösler F, Henriques DYP, Einhäuser W, et al (2010) Online action-to-perception transfer: only percept-dependent action affects perception. Vis Res 50: 2636–2641. 10.1016/j.visres.2010.10.004 20934444

[52] Saygin AP, Wilson SM, Hagler DJ Jr, Bates E, Sereno MI (2004) Point-Light Biological Motion Perception Activates Human Premotor Cortex. J Neurosci 24: 6181–6188. 15240810 10.1523/JNEUROSCI.0504-04.2004 PMC6729669

[53] Saygin AP (2007) Superior temporal and premotor brain areas necessary for biological motion perception. Brain 130: 2452–2461. 17660183 10.1093/brain/awm162

[54] Cohen J (1973) Eta-squared and partial eta-squared in fixed factor ANOVA designs. Educ Psychol Meas 33: 107–12.

[55] Wheatstone C (1838) Contributions to the physiology of vision. Part the First. On some remarkable, and hitherto unobserved, phænomena of binocular vision. Phil Trans R Soc Lon 128: 371–394.

[56] Necker L (1832) Observations on some remarkable optical phenomena seen in Switzerland, and on an optical phenomenon which occurs on viewing a figure of a crystal or geometrical solid. Lond Edinburgh Philos Mag J Sci 1: 329–337.

[57] Hugrass L, Crewther D (2012) Willpower and Conscious Percept: Volitional Switching in Binocular Rivalry. PLoS One 7: 1–9. 10.1371/journal.pone.0035963 PMC3338481 22558283

[58] Logothetis NK, Schall JD (1990) Binocular motion rivalry in macaque monkeys: Eye dominance and tracking eye movements. Vis Res 30: 1409–1419. 2247951 10.1016/0042-6989(90)90022-d

[59] Sun F, Tong J, Yang Q, Tian J, Hung GK (2002) Multi-directional shifts of optokinetic responses to binocular-rivalrous motion stimuli. Brain Res 944: 56–64. 12106665 10.1016/s0006-8993(02)02706-3

[60] Laubrock J, Engbert R, Kliegl R (2008) Fixational eye movements predict the perceived direction of ambiguous apparent motion. J Vis 8: 1–17. 10.1167/8.14.13 19146314

[61] Wohlschläger A (2000) Visual motion priming by invisible actions. Vis Res 40: 925–930. 10720663 10.1016/s0042-6989(99)00239-4

[62] Mitsumatsu H (2009) Voluntary action affects perception of bistable motion display. Perception 38: 1522–1535. 19950483 10.1068/p6298

[63] Cattaneo L, Barchiesi G, Tabarelli D, Arfeller C, Sato M, et al (2010) One's motor performance predictably modulates the understanding of others' actions through adaptation of premotor visuo-motor neurons. Soc Cogn Affect Neurosci 6: 301–310. 21186167 10.1093/scan/nsq099 PMC3110437

[64] Rizzolatti G, Craighero L (2004) The mirror-neuron system. Annu Rev Neurosci 27: 169–92. 15217330 10.1146/annurev.neuro.27.070203.144230

[65] Heiser M, Iacoboni M, Maeda F, Marcus J, Mazziotta JC (2003) The essential role of Broca's area in imitation. Eur J Neurosci 17: 1123–1128. 12653990 10.1046/j.1460-9568.2003.02530.x

[66] Iacoboni M, Woods RP, Brass M, Bekkering H, Mazziotta JC, et al (1999) Cortical mechanisms of human imitation. Science 286: 2526–2528. 10617472 10.1126/science.286.5449.2526

[67] Salomon R, Lim M, Herbelin B, Hesselmann G, Blanke O (2013) Posing for awareness: Proprioception modulates access to visual consciousness in a continuous flash suppression task. J Vis 13: 1–8. 10.1167/13.7.2 23732119

[68] Pulvermüller F (2005) Brain mechanisms linking language and action. Nat Rev Neurosci 6: 576–582. 15959465 10.1038/nrn1706

[69] Glenberg AM, Sato M, Cattaneo L (2008a) Use-induced motor plasticity affects the processing of abstract and concrete language. Curr Biol 18: R290–291. 18397734 10.1016/j.cub.2008.02.036

[70] Glenberg AM, Sato M, Cattaneo L, Riggio L, Palombo D, et al (2008b) Processing abstract language modulates motor system activity. Q J Exp Psychol 61: 905–919. 10.1080/17470210701625550 18470821

[71] Greenwald AG (1970b) A choice reaction time test of ideomotor theory. J Exp Psyc 80: 20–25. 10.1037/h0029960 5482033

[72] Brass M, Bekkering H, Wohlschläger A, Prinz W (2000) Compatibility between Observed and Executed Finger Movements: Comparing Symbolic, Spatial, and Imitative Cues. Brain Cogn 44: 124–143. 11041986 10.1006/brcg.2000.1225

[73] Blake R, Sobel KV, James TW (2004) Neural synergy between kinetic vision and touch. Psychol Sci 15: 397–402. 15147493 10.1111/j.0956-7976.2004.00691.x

[74] Lunghi C, Binda P, Morrone MC (2010) Touch disambiguates rivalrous perception at early stages of visual analysis. Curr Biol 20: R143–144. 20178754 10.1016/j.cub.2009.12.015

[75] van Ee R, van Boxtel JJA, Parker AL, Alais D (2009) Multisensory Congruency as a Mechanism for Attentional Control over Perceptual Selection. J Neurosci 29: 11641–11649. 19759311 10.1523/JNEUROSCI.0873-09.2009 PMC6665778

[76] Zhou W, Jiang Y, He S, Chen D (2010) Olfaction Modulates Visual Perception in Binocular Rivalry. Curr Biol 20: 1356–1358. 20598540 10.1016/j.cub.2010.05.059 PMC4226334

[77] Hommel B (2004) Event files: Feature binding in and across perception and action. Trends Cogn Sci 8: 494–500. 15491903 10.1016/j.tics.2004.08.007

[78] Zhang P, Jamison K, Engel S, He B, He S (2011) Binocular Rivalry Requires Visual Attention. Neuron 71: 362–369. 21791293 10.1016/j.neuron.2011.05.035 PMC3175243

[79] van Ee R, van Dam LCJ, Brouwer GJ (2005) Voluntary control and the dynamics of perceptual bi-stability. Vis Res 45: 41–55. 15571737 10.1016/j.visres.2004.07.030

[80] Paffen CLE, Alais D, Verstraten FAJ (2006) Attention Speeds Binocular Rivalry. Psychol Sci 17: 752–756. 16984290 10.1111/j.1467-9280.2006.01777.x

[81] Ooi TL, He ZJ (1999) Binocular rivalry and visual awareness: the role of attention. Perception 28: 551–574. 10664754 10.1068/p2923

[82] Mitchell JF, Stoner GR, Reynolds JH (2004) Object-based attention determines dominance in binocular rivalry. Nature 429: 410–413. 15164062 10.1038/nature02584

[83] Chong SC, Blake R (2006) Exogenous attention and endogenous attention influence initial dominance in binocular rivalry. Vis Res 46: 1794–1803. 16368126 10.1016/j.visres.2005.10.031

[84] Chong SC, Tadin D, Blake R (2005) Endogenous attention prolongs dominance durations in binocular rivalry. J Vis 5: 1004–1012. 16441198 10.1167/5.11.6

[85] Kanai R, Tsuchiya N, Verstraten FA (2006) The scope and limits of top-down attention in unconscious visual processing. Curr Biol 16: 2332–2336. 17141615 10.1016/j.cub.2006.10.001

[86] Bahrami B, Carmel D, Walsh V, Rees G, Lavie N (2008) Spatial attention can modulate unconscious orientation processing. Perception 37: 1520–1528. 19065856 10.1068/p5999

[87] Rizzolatti G, Riggio L, Dascola I, Umiltà CA (1987) Reorienting attention across the horizontal and vertical meridians - Evidence in favour of a premotor theory of attention. Neuropsychologia 25: 31–40. 3574648 10.1016/0028-3932(87)90041-8

[88] Smith DT, Schenk T (2012) The premotor theory of attention: time to move on? Neuropsychologia 50 (6): 1104–1114. 22306518 10.1016/j.neuropsychologia.2012.01.025

[89] Fang F, He S (2005) Cortical responses to invisibile objects in the human dorsal and ventral pathways. Nat Neurosci 8 (10): 1380–1385. 16136038 10.1038/nn1537

